# Evaluation of Gene, Protein and Neurotrophin Expression in the Brain of Mice Exposed to Space Environment for 91 Days

**DOI:** 10.1371/journal.pone.0040112

**Published:** 2012-07-09

**Authors:** Daniela Santucci, Fuminori Kawano, Takashi Ohira, Masahiro Terada, Naoya Nakai, Nadia Francia, Enrico Alleva, Luigi Aloe, Toshimasa Ochiai, Ranieri Cancedda, Katsumasa Goto, Yoshinobu Ohira

**Affiliations:** 1 Behavioural Neuroscience Section, Cellular Biology and Neuroscience Department, Istituto Superiore di Sanità, Rome, Italy; 2 Graduate School of Medicine, Osaka University, Osaka, Japan; 3 Graduate School of Frontier Biosciences, Osaka University, Osaka, Japan; 4 Japan Aerospace Exploration Agency, Ibaraki, Japan; 5 Institute of Neurobiology and Molecular Medicine, CNR, European Brain Research Institute, Rome, Italy; 6 Mitsubishi Heavy Industries, Hyogo, Japan; 7 DOBIG, University of Genova, Genova, Italy; 8 Graduate School of Health Sciences, Toyohashi SOZO University, Aichi, Japan; Pennington Biomedical Research Center, United States of America

## Abstract

Effects of 3-month exposure to microgravity environment on the expression of genes and proteins in mouse brain were studied. Moreover, responses of neurobiological parameters, nerve growth factor (NGF) and brain derived neurotrophic factor (BDNF), were also evaluated in the cerebellum, hippocampus, cortex, and adrenal glands. Spaceflight-related changes in gene and protein expression were observed. Biological processes of the up-regulated genes were related to the immune response, metabolic process, and/or inflammatory response. Changes of cellular components involving in microsome and vesicular fraction were also noted. Molecular function categories were related to various enzyme activities. The biological processes in the down-regulated genes were related to various metabolic and catabolic processes. Cellular components were related to cytoplasm and mitochondrion. The down-regulated molecular functions were related to catalytic and oxidoreductase activities. Up-regulation of 28 proteins was seen following spaceflight vs. those in ground control. These proteins were related to mitochondrial metabolism, synthesis and hydrolysis of ATP, calcium/calmodulin metabolism, nervous system, and transport of proteins and/or amino acids. Down-regulated proteins were related to mitochondrial metabolism. Expression of NGF in hippocampus, cortex, and adrenal gland of wild type animal tended to decrease following spaceflight. As for pleiotrophin transgenic mice, spaceflight-related reduction of NGF occured only in adrenal gland. Consistent trends between various portions of brain and adrenal gland were not observed in the responses of BDNF to spaceflight. Although exposure to real microgravity influenced the expression of a number of genes and proteins in the brain that have been shown to be involved in a wide spectrum of biological function, it is still unclear how the functional properties of brain were influenced by 3-month exposure to microgravity.

## Introduction

Altered gravitational environment represents a unique challenge for biological systems that have evolved against a constant gravitational background and it has been reported that exposure to actual and/or simulated microgravity, as well as to rotationally induced hypergravity, causes various physiological adaptations, including in the central nervous system (CNS) [1]–[4] and antigravity muscle [5], [6] in mice, rats and humans. For example, 16 days of spaceflight caused a change in the synaptic circuitry at the hindlimb cortex of the postnatal developing rats [2], while exposure of rats to hypergravity from day 11 of gestation to postnatal day 15 led to substantial and persistent delay in the development of the cortical monaminergic projections to the spinal cord in young rats [7] or to altered cerebellar growth [8].

In addition, rats flown on the space shuttle (Neurolab) from postnatal day 8 to 24 in 1998 showed an abnormal development of extensor motoneurons and changes in the number and morphology of cortical synapses [9]. Analogously, exposure to hypergravity affects exploratory behavior and ability to discriminate a new spatial arrangement [10], [11], as well as nerve growth factor (NGF) and brain derived neurotrophic factor (BDNF) levels, in the CNS of mice [12], [13]. Moreover, modulation of genes coding for proteins involved in a wide range of cellular functions (DNA/RNA metabolism, protein processing, intermediate metabolism, cytoskeleton and motility, cell cycle and apoptosis, signal transduction, and neuronal structure/function) has been seen in the brain of adult mice exposed to acute 2-G hypergravity [14].

In particular, in terms of the nervous system, the hindlimb suspension, which is often used as the simulation model for exposure to microgravity environment, causes the decrease of electromyogram activity in soleus muscle, afferent neurograms in spinal cord [15], GABAergic neurons in the hindlimb somatosensory cortex [1], and neurogenesis in rats [16]. Recently, proteomic analyses of the hippocampus [17] and the hypothalamus [18], as well as a microarray analysis of gene expression in mouse brain [19], were performed to elucidate the mechanism responsible for the adaptation to gravitational unloading. After 7 days of hindlimb suspension, the expression of cytoskeletal proteins, such as tubulin and metabolic proteins in hippocampus of adult mice changed. Seven spots were decreased and 4 spots were increased [17], and biomarkers of oxidative stress in hypothalamus of mice were increased [18]. Therefore, exposure to simulated microgravity environments by hindlimb suspension might also induce distinct changes specific to the regions of the brain.

However, it is not clear how the characteristics of mouse brain respond to long-term inhibition of antigravity activity. Therefore, the current study was performed to investigate the effects of long-term exposure to microgravity environment on the characteristics of brain in mice, since we had the access to brain samples by participating in the “tissue sharing team” [20]. Specifically, comprehensive analyses of gene and protein expression were performed. Further, responses of NGF and BDNF in brain, as well as adrenal glands where the level of neutrophin expression is also regulated [21], were investigated, because the changes in neurotrophin levels in CNS following exposure to challenging environment and the roles of these neurobiological determinants were also reported [22].

## Materials and Methods

### Experimental Design and Animal Care

The protocol utilized in the study has been authorized by the Public Veterinary Health Department of the Italian Ministry of Health. The experimental procedures were also conducted in accordance with the *Guide for the Care and Use of Laboratory Animals* of the Japanese Physiological Society, NIH *Guide for the Care and Use of Laboratory Animals* and following recommendations reported in European Communities Council Directive of 24 November 1986 (86/609/EEC). This study was also approved by the Committee on Animal Care and Use at Graduate School of Medicine, Osaka University (No. 22-071).

The spaceflight experiments were carried out using male C57BL/10J mice (8 weeks old at launch). Wild type and pleiotrophin transgenic mice (n = 3 each) were individually housed in mouse drawer system (MDS, 11.6×9.8×8.4 cm), which is a payload developed by Alenia-Space [23]. Pleiotrophin transgenic mice were utilized to investigate the possibility of this osteogenic factor for protection of osteoporosis [24]. Food and water were supplied *ad libitum*. These mice were launched by space shuttle “Discovery” (space transport system, STS, -128) on August 28, 2009. They were housed in Japanese Experimental Module (Kibo) on the International Space station (ISS) until they returned to the Earth by space shuttle “Atlantis” (STS-129) on November 27, 2009. Only 1 wild type and 2 transgenic mice returned to the Earth alive after 91 days of flight. Whole brain and adrenal glands were sampled from each mouse sacrificed by inhalation of carbon dioxide at the Life Science Support Facility of Kennedy Space Center within 3 hours after landing.

After the spaceflight experiment, on-ground experiment was also carried out at the Vivarium of the Advanced Biotechnology Center in Genova, Italy. One group of mice with the same species, sex, and age were housed in MDS for 3 months as the ground controls (GCs). Another group of mice were housed in normal vivarium cage as the laboratory controls (LCs). Amount of food and water supplementation and environmental conditions were simulated as the flight group [20]. After 3 months, brain was sampled from 1 wild type and 2 transgenic mice housed in MDS (GC) as was stated above. Further, samples were also obtained from group LC (n = 3 in each genotypes). The brain was cut into two portions longitudinally. And both right and left side of brain were immediately frozen in liquid nitrogen and stored at −80°C until analyses. The adrenal glands were also saved equally.

**Figure 1:**
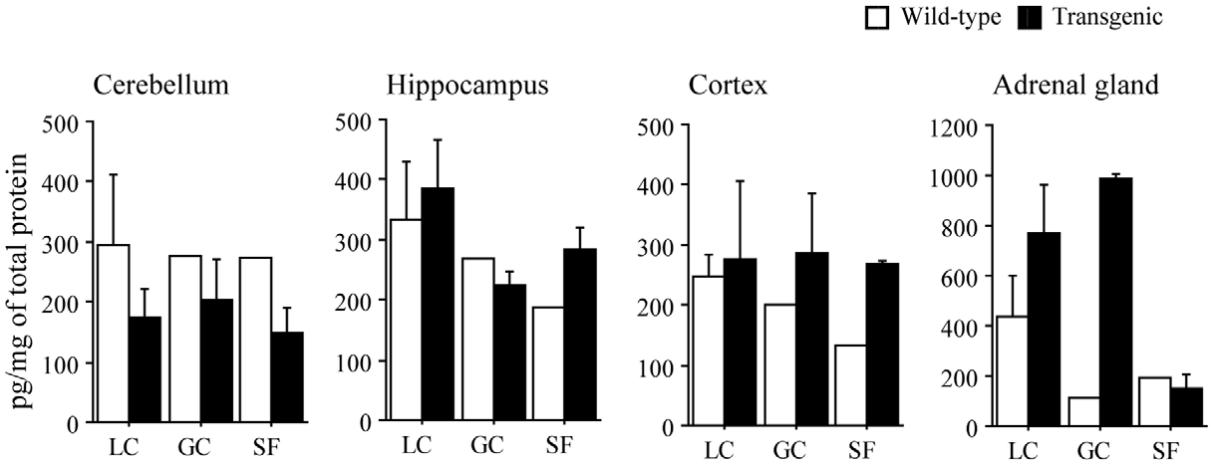
Responses of nerve growth factor in brain and adrenal gland. LC, GC, and SF: laboratory control, ground control, and spaceflight group, respectively. The numbers of wild type mice in LC, GC, and SF are 3, 1, and 1. Those of transgenic mice are 3, 2, and 2. Mean±SEM.

**Figure 2:**
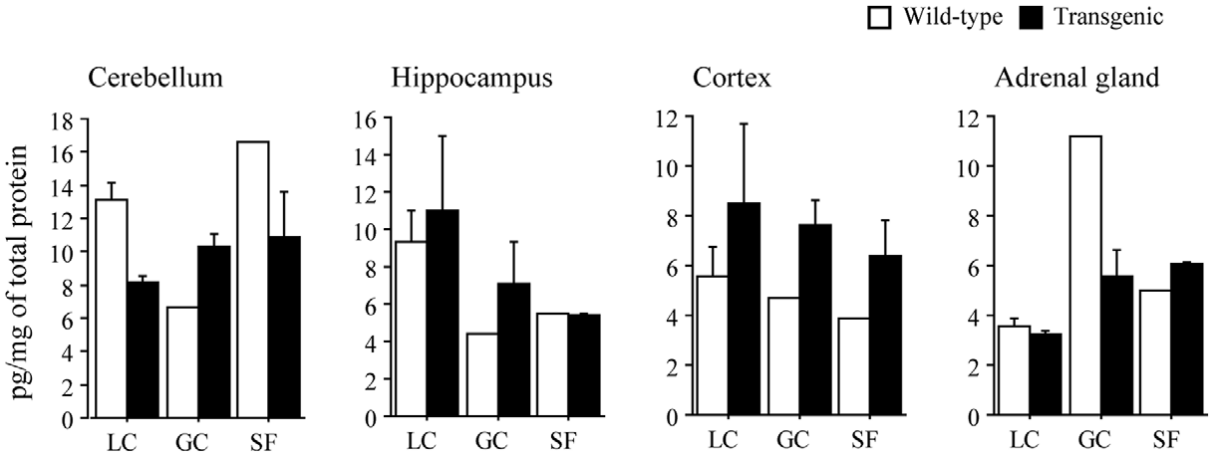
Responses of brain derived neurotrophic factor in brain and adrenal gland. See Figure 1 for the abbreviations and number of mice in each group. Mean±SEM.

### Neurotrophin Assays

Neurobiological parameters, known to be involved in the response to stress, have been evaluated in the left brain and adrenal gland. In particular, NGF levels have been measured in the cerebellum, hippocampus, cortex, and adrenal glands, as was described previously [25]. The levels of NGF were measured by a commercially available kit (Promega Italia, Milano, Italy). Briefly, polystyrene 96-well microtube immunoplates (Nunc) were coated with affinity purified polyclonal goat anti-NGF antibody, diluted in 0.05 M carbonate buffer (pH 9.6). Parallel wells were coated with purified goat IgG (Zymed, San Francisco, CA, U.S.A.) in order to evaluate the non-specific signal. Following an overnight incubation at room temperature and 2-hour incubation with a blocking buffer {0.05 M carbonate buffer with 1% bovine serum albumin (BSA), pH 9.5}, plates were washed three times with 50 mM Tris-HCl, 200 mM NaCl, 0.5% gelatin, and 0.1% Triton X-100 (pH 7.4). After extensive washing of the plates, the samples and the NGF standard solutions were diluted with sample buffer {0.1% Triton X-100, 100 mM Tris-HCl, 400 mM NaCl, 4 mM ethylenediaminetetraacetic acid (EDTA), 0.2 mM phenylmethanesulfonyl fluoride (PMSF), 0.2 mM benzethonium chloride, 2 mM benzamidine, 40 U/ml aprotinin, 0.05% sodium azide, 2% BSA and 0.5% gelatine, pH 7.2}, distributed among the wells and left to stand at room temperature overnight. The plates were then washed three times and incubated with anti-ß-NGF-galactosidase (Boehringer Mannheim, Germany, 4 mU/well) at 37°C for 2 hours. After further washing, 100 µL of substrate buffer {4 mg/mL of chlorophenol red, 100 mM 4-(2-hydroxyethyl)-1-piperazineethanesulfonic acid) (HEPES), 150 mM NaCl, 2 mM MgCl_2_, 0.1% sodium azide, and 1% BSA} were added to each well. After 2-hour-incubation at 37°C, optical density was measured using an ELISA reader (MR 5000, Dynatech, Denkendorf, Germany) at 575 nm. The values of standards and samples were corrected by considering the non-specific binding level. Under these conditions, the sensitivity was 3 pg/mL. The recovery of NGF in our assay ranged from 80 to 90% [26], [27] and cross-reactivity with other molecules of the NGF family, such as NT-3 and NT-4/5, was less than 3%. Data were represented as pg/mg of total protein.

**Figure 3:**
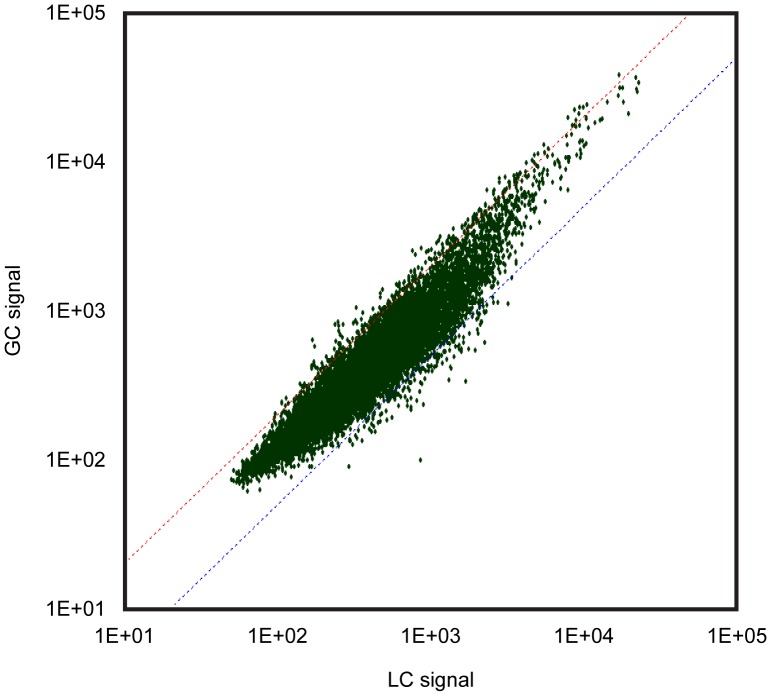
Scatter plots showing the relationship of gene expression between group LC and GC. The dots above and below the dotted slanted lines show the genes in group GC up- and down-regulated, more than 2 folds and/or less than half, vs. those in group LC, according to SAM analysis. See Figure 1 for the abbreviations.

**Figure 4:**
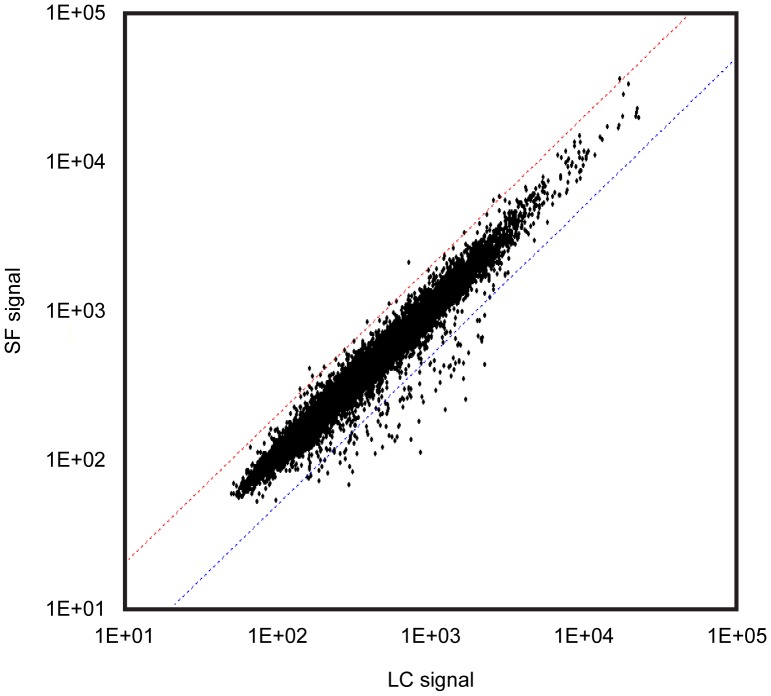
Scatter plots showing the relationship of gene expression between group LC and SF. The dots above and below the dotted slanted lines show the genes in group SF up- and down-regulated, more than 2 folds and/or less than half, vs. those in group GC, according to SAM analysis. See Figure 1 for the abbreviations.

**Figure 5:**
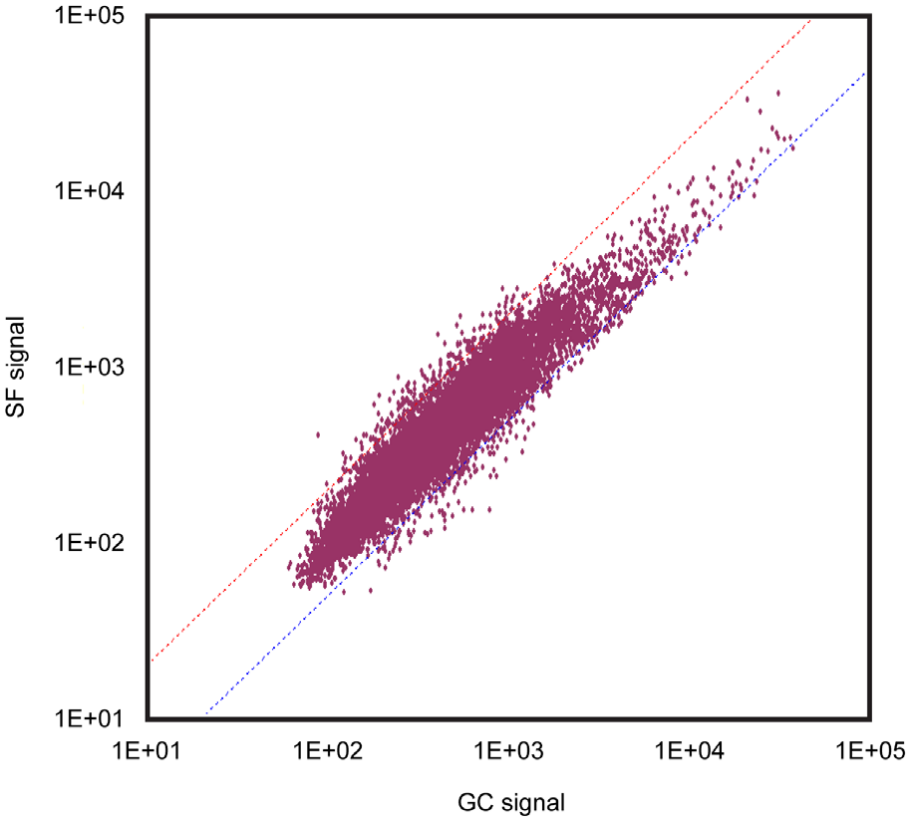
Scatter plots showing the relationship of gene expression between group GC and SF. The dots above and below the dotted slanted lines show the genes in group SF up- and down-regulated, more than 2 folds and/or less than half, vs. those in group GC, according to SAM analysis. See Figure 1 for the abbreviations.

Quantification of endogenous BDNF was performed by using two-site enzyme immunoassay kit (Promega Italia, Milano, Italy). 96-well immunoplates (Nunc) were coated with monoclonal anti-mouse-BDNF antibody (100 µL per well) and incubated overnight at 4°C. Then the plates were washed three times with wash buffer and the samples were incubated in the coated wells (100 µL each) for 2 hours at room temperature with shaking. After additional washes, the antigen was incubated an anti-human BDNF antibody for 2 hours at room temperature with shaking. The plates were washed again with wash buffer and then incubated with an anti-IgY HRP for 1 hour at room temperature. After another wash, the plates were incubated with a TMB/peroxidise substrate solution for 15 minutes. And 1 M phosphoric acid (100 µL/well) was added to the wells. The colorimetric reaction product was measured at 450 nm using an ELISA reader (Dynatech MR 5000, Dynatech, Denkendorf, Germany). BDNF concentrations were determined from the regression line for the BDNF standard (ranging from 7.8 to 500 pg/mL-purified mouse BDNF) incubated under similar conditions in each assay. The sensitivity of the assay was 15 pg/mL of BDNF and the cross-reactivity with other related neurotrophic factors, e.g. NGF, NT-3, andNT-4, was less than 3% [28]. BDNF concentration was expressed as pg/mg of total protein and all assays were performed in triplicate.

**Table 1:** Statistically significant GO terms derived from up-regulated genes (vs. ground control).

ID	GO Term	p-value
	Biological process	
0006952	defense response	5.14E-05
0006955	immune response	5.27E-06
0002376	immune system process	1.15E-05
0006954	inflammatory response	1.60E-05
0008152	metabolic process	1.33E-05
0055114	oxidation-reduction process	1.14E-05
0042035	regulation of cytokine biosynthetic process	3.14E-05
0042108	positive regulation of cytokine biosynthetic process	8.55E-05
0045402	regulation of interleukin-4 biosynthetic process	1.26E-04
0045404	positive regulation of interleukin-4 biosynthetic process	1.26E-04
0006950	response to stress	3.21E-05
0009611	response to wounding	8.06E-05
	Cellular component	
0005792	microsome	6.12E-05
0042598	vesicular fraction	6.93E-05
	Molecular function	
0003824	catalytic activity	8.57E-06
0005125	cytokine activity	1.24E-04
0004497	monooxygenase activity	1.41E-05
0016491	oxidoreductase activity	1.44E-06
0016705	oxidoreductase activity, acting on paired donors, with incorporation or reduction of molecular oxygen	2.61E-05

**Table 2:** Statistically significant GO terms derived from down-regulated genes (vs. ground control).

GO ID	GO Term	p-value
	Biological process	
0006066	alcohol metabolic process	3.77E-06
0005975	carbohydrate metabolic process	9.50E-06
0044262	cellular carbohydrate metabolic process	3.74E-07
0044237	cellular metabolic process	4.35E-09
0009987	cellular process	2.45E-06
0006091	generation of precursor metabolites and energy	1.11E-08
0006007	glucose catabolic process	1.51E-05
0006006	glucose metabolic process	2.94E-06
0019320	hexose catabolic process	1.51E-05
0019318	hexose metabolic process	1.27E-06
0008152	metabolic process	2.98E-08
0046365	monosaccharide catabolic process	1.51E-05
0005996	monosaccharide metabolic process	3.47E-06
0055114	oxidation-reduction process	4.29E-10
0044238	primary metabolic process	9.38E-06
0044283	small molecule biosynthetic process	4.03E-06
0044281	small molecule metabolic process	3.91E-11
	Cellular component	
0005737	cytoplasm	1.47E-10
0044444	cytoplasmic part	2.21E-08
0031975	envelope	1.09E-06
0005622	intracellular	6.25E-06
0044446	intracellular organelle part	2.17E-05
0044424	intracellular part	1.51E-06
0005740	mitochondrial envelope	5.54E-08
0005743	mitochondrial inner membrane	2.75E-08
0031966	mitochondrial membrane	3.63E-08
0044429	mitochondrial part	2.16E-07
0005739	mitochondrion	3.82E-09
0031967	organelle envelope	1.03E-06
0019866	organelle inner membrane	3.82E-08
0031090	organelle membrane	1.99E-06
0070469	respiratory chain	1.05E-07
	Molecular function	
0003824	catalytic activity	3.18E-07
0016491	oxidoreductase activity	4.06E-06

### Analyses of Genes and Proteins

Comprehensive analyses of gene and protein expression were performed in the right brain. Each of the frozen samples was individually put into a mortar, and then powdered by pestle-grinding in liquid nitrogen. For each sample, one half of the powdered frozen tissue without liquid nitrogen (evaporated) was kept in an Eppendorf tube for gene expression analysis. And the remaining half was also kept in another Eppendorf tube for analyses of protein expression. Only the data obtained from wild type mice were utilized in order to investigate the effects of spaceflight on the expression levels of genes and proteins.

#### Gene expression analysis using DNA microarray

Total RNA was extracted and purified from the whole brain of the tested mice (all of the tissue sections were mixed and powdered in liquid nitrogen) using RNeasy Mini kit (QIAGEN, Inc, Valencia, CA, U.S.A.). The purified total RNA samples were assessed for their quantity using Agilent 2100 Bioanalyzer with RNA 6000 Nano Assay kit (Agilent Technologies, Inc., Santa Clara, CA, USA) and ultraviolet spectrophotometer. Preparation of Cy5-labeled amino allyl aRNA from the total RNA was performed according to the instruction manual of Amino Allyl MessageAmp™ II aRNA Amplification kit (Ambion Inc., Austin, TX, USA). Briefly, 1 µg each of the purified total RNA was reverse-transcribed with T7-oligo dT primer to synthesize single-stranded cDNA (ss-cDNA). Subsequently, double-stranded cDNA was synthesized from the ss-cDNA, and *in vitro* transcription with amino-allyl UTP to generate amino-allyl labeled aRNA target sample was performed. The aRNA samples were purified and coupled with amine reactive Cy5 fluorescent dye, then Cy5-coupled aRNA samples were spin column-purified (RNeasy Mini) for the microarray hybridization experiment.

Mouse EG4000 oligo DNA microarray used for this study was designed and fabricated by CombiMatrix B3 CustomArray™ synthesizer at Ecogenomics, Inc. (Kurume City, Fukuoka, Japan), and it contained triplicate 40-mer oligo DNA probes representing 3,998 *Mus musculus* RefSeq cDNAs, that were archived in National Center Biotechnology Information (NCBI) GenBank database. The detailed information regarding the probes and the template cDNAs can be accessed via the NCBI Gene Expression Omnibus (GEO) accession number of GSE32077, or at the following web site, http://www.ncbi.nlm.nih.gov/geo.

**Table 3:** Up-regulated proteins due to spaceflight (vs. ground control).

Name of gene	Name of protein	Fold change
Ap2a2	AP-2 complex subunit alpha-2	1.74
Atp1a1	Sodium/potassium-transporting ATPase subunit alpha-1	1.67
Atp2a2	Isoform SERCA2B of Sarcoplasmic/endoplasmic reticulum calcium ATPase 2	2.88
Atp5a1	ATP synthase subunit alpha, mitochondrial	2.47
Atp6v0a1	ATPase, H+ transporting, lysosomal V0 subunit a isoform 1	1.50
Atp6v1h	V-type proton ATPase subunit H	1.91
Atpif1	ATPase inhibitor, mitochondrial precursor	1.77
Basp1	Brain acid soluble protein 1	1.60
Calb2	Calretinin	1.96
Camk2a	Isoform Alpha CaMKII of Calcium/calmodulin- dependent protein kinase type II subunit alpha	2.78
Camkv	CaM kinase-like vesicle-associated protein	1.67
Cltc	Clathrin, heavy polypeptide	1.63
Cntn1	Contactin-1	2.00
Cox5a	Cytochrome c oxidase subunit 5A, mitochondrial	2.44
Crmp1	Crmp1 protein	1.66
Dnm1	Isoform 3 of Dynamin-1	2.73
Dpysl2	Dihydropyrimidinase-related protein 2	1.19
mt-Co2	Cytochrome c oxidase subunit 2	3.50
Ppp3ca	Isoform 1 of Serine/threonine-protein phosphatase 2B catalytic subunit alpha isoform	1.41
Pvalb	Parvalbumin alpha	2.07
Sept7	Septin-7	1.33
Slc1a2	Isoform Glt-1 of Excitatory amino acid transporter 2	2.19
Slc25a12	Calcium-binding mitochondrial carrier protein Aralar1	3.98
Snap25	Isoform SNAP-25b of Synaptosomal-associated protein 25	1.51
Stx1b	Syntaxin-1B	1.37
Sucla2	Succinyl-CoA ligase [ADP-forming] subunit beta, mitochondrial	1.47
Syn1	Isoform Ib of Synapsin-1	1.51
Uqcrc1	Cytochrome b-c1 complex subunit 1, mitochondrial precursor	3.50

**Table 4:** Down-regulated proteins due to spaceflight (vs. ground control).

Name of gene	Name of protein	Fold change
Abat	Isoform 1 of 4-aminobutyrate aminotransferase, mitochondrial	0.63
Acot7	Isoform C of Cytosolic acyl coenzyme A thioester hydrolase	0.69
Alb	Serum albumin	0.71
Aldoa	Fructose-bisphosphate aldolase A isoform 1	0.74
Car2	Carbonic anhydrase 2	0.55
Cnp	Isoform CNPII of 2',3'-cyclic-nucleotide 3'-phosphodiesterase	0.53
Cycs	Cytochrome c, somatic	0.45
Glud1	Glutamate dehydrogenase 1, mitochondrial	0.49
Pebp1	Phosphatidylethanolamine-binding protein 1	0.98

The Cy5-labeled aRNA target samples were then hybridized with the probes on microarrays (one microarray hybridization per sample, three samples in each of the three exposure condition), and total of 9 microarrays were used for this experiment. The hybridization was carried out for 16 hours at 45°C in 100 µl of 25% formamide/6x SSPE/0.04% SDS/0.05% Tween-20/20 mM EDTA solution, followed by post-hybridization washes (two washes in 6x SSPE/0.05% Tween-20 at 45°C for 5 minutes each, two washes in 3x SSPE/0.05% Tween-20 at ambient temperature for 1 minute each, two washes in 0.5x SSPE/0.05% Tween-20 at ambient temperature for 1 minute each, two washes in 2x PBS/0.1% Tween-20 at ambient temperature for 1 minute each, and two final washes in 2x PBS at ambient temperature for 1 minute each). The post-hybridization wash process-completed microarray slides were scanned with GenePix 4000B scanner (Molecular Devices, Sunnyvale, CA, USA) at 5 µm resolution in order to obtain quantified gene expression data sets. Microarray data are accessible through GEO with accession number of GSE32077.

Data preprocessing and analysis were performed using GeneSpring software 11.0.1 (Agilent Technologies). A preprocess procedure were performed according to the manufacturer’s recommendations and MicroArray Quality Control project reports [29]. Briefly, a decision-matrix determines whether each transcript is reliably detected (ie, present), marginally detected (ie, marginal), or not detected (ie, absent), and calculates signal intensities. Normalization was carried out to the 50th percentile of each array, and each gene to the median, by choosing the GeneSpring normalization option. In terms of the enriched gene ontology (GO) terms and pathway analysis, the normalized logarithmic intensity ratios were used with 3 selective filters to remove data with a poor signal to noise ratio. Those genes were filtered out with a raw signal intensity <100. Genes with <2-fold changes were then removed. Finally, t-test was used to achieve a more stringent selection of the normalized log-transformed intensity ratios at each time point vs. the control sample. The probabilities were 0.1 adjusted by the false-discovery rate for corrections of multiple tests.

Statistical analyses: The levels of gene expression were compared between each group. Genes with significant up-regulation (more than 2 folds) and down-regulation (less than half) were identified. The scanned and quantified gene expression data were statistically analyzed using Significance Analysis of Microarray (SAM) at locally-determined false discovery rate [30]–[33]. For each of the genes (gene probes) on the DNA microarray, differential gene expression was considered significant, when the effective false discovery rate (q-value) was below the lowest false discovery rate as calculated by permutations of gene-specific test in SAM. SAM-p, which shows the t-statistics value calculated by SAM and significance (p<0.05) in differential gene expression, as well as Fold-Difference (FD) in up- or down-regulation between each group (GC vs. LC, SF vs. GC, and SF vs. LC), were examined using the two-class paired response type of SAM. All of the significant DNA microarray data obtained in this study were deposited in the NCBI’s GEO, and they are accessible through GEO Series accession number GSE32077.

#### Protein expression analyses

The commercial iTRAQ**®** analysis serves (Filgen) was utilized for the mass spectrometric analysis. Briefly, the frozen powder was dissolved in the Tissue Protein Extraction Reagent (PIERCE). The proteins (100 µg) containing in the extract was digested by trypsin for 24 hours at 37°C. The iTRAQ**®** reporter with different molecular weight was conjugated in each group using iTRAQ**®** reagent-multiplex assay kit and multiplex buffer kit (AB SCIEX) for 2 hours at 25°C. The conjugated peptide fragments in group SF, GC and LC were combined, separated into eight fractions using Caution Exchange Buffer Pack (AB SCIEX), and desalted by Sep-Pak**®** Light C18 Cartridge (Waters). Subsequently, the mass spectrometric analysis was performed using QSTAR**®** Elite Hybrid LC/MS/MS system (AB SCIEX). The peaks obtained in the mass spectrum were further analyzed by the MS/MS measurement. The quantitative comparison was made using the peak level of the reporter molecule, which was seen in the MS/MS spectrum. The database search on the MCBI web site was performed using the obtained mass and sequence of peptides.

Statistical analysis: The peptides, which were matched within the same protein, were pooled and the level in each peak of reporter was considered as the individual value. The significant difference was examined by unpaired t-test. Differences were considered significant at the 0.1 level of confidence.

## Results

### Responses of NGF and BDNF

Despite the lack of a sufficient number of animals and tissues available for a complete statistical evaluation of the effect of 91-day-exposure to space environment, the expression of NGF in hippocampus and cortex of the spaceflight wild type animal tended to be less than ground controls (LC and GC, Fig. 1). The level in adrenal gland was also lower than group LC (p>0.05). The level in group GC was also lower similarly. The mean level in cerebellum was identical between 3 groups. As for the transgenic mice, spaceflight-related reduction of NGF occurred only in adrenal gland. Consistent trends between various portions of brain and adrenal gland were not observed in the responses of BDNF to spaceflight (Fig. 2). Although the mean levels in cerebellum and adrenal gland of SF-transgenic mice, as well as GC-transgenic, tended to be greater than in LC, those trends in hippocampus and cortex were minor. However, response of BDNF in each organ of wild type mouse to spaceflight was not clear, since only one mouse came back to the Earth alive.

### Responses of Genes

Totally 4000 genes were analyzed. Figures 3, 4, and 5 show the relationship of gene expression between each group. More genes tended to be down-regulated in response housing in MDS either on the Earth (Fig. 3) or in space (Fig. 4) vs. the ground-based group, LC, housed in the regular vivarium cage. The numbers of genes up- and down-regulated in response spaceflight, more than 2 folds and less than half vs. group GC, were 125and 117, respectively (Fig. 5, Table S1 and S2).

Statistically significant GO terms from up and down-regulated genes are shown in Table 1 and 2, respectively. Among GO terms of up-regulated genes, several enriched terms in biological process categories were generally related to the immune/inflammatory and metabolic process (Table 1). And the categories for cellular component contained microsome and vesicular fraction. Molecular function categories were related to various enzyme activities. In GO terms of down-regulated genes, biological process contained glucose/carbohydrate metabolism (Table 2). Cellular components were related to mitochondrial, intracellular, cytoplasmic, and organelle properties. The down-regulated molecular functions were related to catalytic and oxidoreductase activities.

### Responses of Proteins

Totally 776 proteins were identified in the mass spectrometric analysis. Table 3 shows 28 proteins up-regulated in response to spaceflight vs. those in ground control. Seven proteins, such as ATP synthase subunit alpha, cytochrome c oxidase subunit 5A, cytochrome c oxidase subunit 2, and cytochrome b-c1 complex subunit 1, were related to mitochondrial metabolism. Some proteins, which involve in the synthesis and hydrolysis of ATP, were also up-regulated. Further, expressions of 6 proteins, which play some roles in calcium/calmodulin metabolism, were elevated. Eight proteins were related to nervous system, such as long-term potentiation and/or neurotransmitter release (CaMKII of calcium/calmodulin-dependent protein kinase type II subunit alpha, SNAP-25b of synaptosomal-associated protein 25, and isoform Ib of synapsin-1) and development of nervous system (contactin-1, dihydropyrimidinase-related protein 2). Furthermore, proteins related to transport of proteins and/or amino acids, such as AP-2 complex subunit alpha-2, clathrin heavy polypeptide, were also up-regulated in response to spaceflight. Spaceflight-related down-regulation was noted in 9 proteins (Table 4). Many of them, such as cytochrome c and glutamate dehydrogenase, were related to mitochondrial metabolism.

## Discussion

Responses of gene and protein expression to 3-month spaceflight were studied in mouse brain. Further, two proteins, NGF and BDNF, which are involved in learning and memory performances, ageing-related disorders and anxiety-like behavior, were also analyzed in brain and adrenal gland. Although a small number of animals was available for this study, data suggested that exposure to real microgravity influenced the expression of a number of genes and proteins in the brain that have been shown to be involved in a wide spectrum of biological function.

Significant overlapping up- or down-regulations of gene and protein in response to spaceflight were not observed in most of the parameters. However, an overlapping spaceflight-related down-regulation of gene and protein expression was seen in carbonic anhydrase 2 (Table 4 and Table S2). Further, cytochrome c (somatic) protein (Table 4) and cytochrome c-1 (Cyc1) gene (Table S2) were also down-regulated. These results may suggest a close functional relationship between genes and proteins.

However, disagreement of the responses between genes and proteins was also noted. Although down-regulation of biological process was seen in the genes involved in the metabolic and oxidation-reduction process (Table 2), 7 proteins, such as ATP synthase subunit alpha, cytochrome c oxidase subunit 5A, cytochrome c oxidase subunit 2, and cytochrome b-c1 complex subunit 1, related to mitochondrial metabolism were up-regulated (Table 3). It is speculated that these genes may be also up-regulated during the early phase of spaceflight, since protein synthesis follows the status of gene expression generally. The expression of these genes could be down-regulated, once the up-regulation of proteins was completed. However, time-course changes in the expression of genes and proteins were not possible in the present experiment unfortunately.

It was found that among those significantly up-regulated specifically by spaceflight, several proteins such as CRMP1 encodes for proteins expressed exclusively in the nervous system and implicated in axon guidance, neuronal growth, cone collapse and cell migration [34], while Septin 7 appears to be critical for dendrite branching and dendritic-spine morphology [35]. Genetic evidence that the alpha isoform of calcineurin is important for the reversal of long-term potentiation in the hippocampus having specific functions in modulating neuronal activity in particular cell types has also been reported [36].

Parvalbumin, structurally and functionally similar to calmodulin and troponin C, is thought to be involved in relaxation after contraction in muscle and is expressed in a specific population of GABAergic interneurones, which are believed to have a role in maintaining the balance between excitation and inhibition in the cortex, as well as the hippocampus [37], [38]. Moreover, synapsin I protein is implicated in synaptogenesis and modulation of neurotransmitter release, suggesting a potential role in several neuropsychiatric diseases [39]. By contrast, among the proteins down-regulated by spaceflight, serum albumin is of particular interest, since it has been reported to be essential for maintaining the osmotic pressure needed for proper distribution of body fluids between intravascular compartments and body tissues and functions primarily as a carrier protein for steroids, fatty acids, and thyroid hormones playing a role in stabilizing extracellular fluid volume. Thus, such down-regulation could be related to compensatory mechanisms to maintain the appropriate blood pressure and fluid distribution in space.

Changes in neurotrophin levels in the CNS upon exposure to rotationally induced hypergravity, as well as altered cerebellar growth, have been reported [8], [11]–[13]. A number of studies have shown that NGF and BDNF, in addition to their classic trophic function on neuronal survival and differentiation, act as a modulator of synaptic plasticity [22], [40] influencing the remodelling of nerve terminals, neurotrasmitter and neuropeptide synthesis and release [41], [42] and playing a role in some critical aspects of the CNS plastic response to challenging environment. Both cortical neurons and adrenal gland cells, known to produce and to be receptive to NGF and BDNF, are particularly affected by microgravity, probably by over exposure to three-dimensional motor activity and stress related events. However, it is not clear why the NGF protein levels in the cortex (only in wild type mouse) and adrenal glands of SF mice were reduced. NGF is known to be up-regulated under stress [43], [44] and it is highly possible that a consistent production of both NGF and BDNF in response to continues microgravity exposure is needed by cortical and adrenal gland cells. For example, adrenal gland cells are biological targets of NGF and the reduction in NGF levels observed in the SF mice may suggest “adrenal fatigue” after long-term stimulation due to the 3-month permanence in space. A similar mechanism can take place with cortical cells. Behavioral observations, indicating an increased time spent for “energy saving” profile in these mice [20], seem to support this hypothesis.

The adrenal glands are morphologically (and functionally) endowed with a high degree of plasticity [45]. Their sizes vary according to the short- and medium-term life stressful events and such a morpho-functional change is rapidly reflected in subtle changes; for example, in the expression of the hippocampal glucocorticoid receptor system or in the modulation of a number of hippocampal events, such as the excitability of hippocampal neurons, neurogenesis and synaptogenesis [46].

Expression of NGF in hippocampus, cortex, and adrenal gland of wild type animal tended to decrease following spaceflight. As for pleiotrophin transgenic mice, spaceflight-related reduction of NGF occured only in adrenal gland. Consistent trends between various portions of brain and adrenal gland were not observed in the responses of BDNF to spaceflight. It is not clear why NGF and BDNF that are expressed in the brain and adrenal glands and known to play critical roles in these tissues were not affected remarkably by spaceflight. One reasonable possibility might be that down- and/or up-regulation of NGF and BDNF in response to environmental changes or to stress-related events may not be long-lasting phenomena, but transient responses. For example, the half-life of NGF and BDNF presence in the bloodstream after stress or following exogenous neurotrophin administration is rather short. It is therefore possible that the differences in NGF and BDNF release in response to spaceflight took places during the early exposure, but significant differences are not measurable during the later time points, probably due to the bindings to receptor by NGF target cells. It would be of interest to explore, at ultra-structural levels, whether differences of synaptic neuroplasticity and endocrine response took place in specific NGF and BDNF-responsive forebrain neurons that are involved in learning and memory and in mechanisms or adrenal gland activation. Moreover, it is interesting to study how cortcosteroid receptors can be modulated by long-term exposure to microgravity and how this can, in turn, affect the viability, arborization, and/or number of neurons in the cortex, hippocampus and cerebellum, since the level of glucocorticoid is influenced by stress [47].

The CRMP1 protein in the SF group was up-regulated vs. group GC. Since it was reported that mice with CRMP1 deficiency exhibited impaired spatial learning and memory [48], it is worth mentioning that learning and memory performances may be improved in response to spaceflight. Several other studies [14], [19] have been specifically focused on the changes in hippocampal gene expression following simulated microgravity or hypergravity and reported that alteration in gravity vector might affect several aspects of the hippocampal function (structural and functional) in order to compensate for environmental changes. Oke et al. [49] recently reported that hindlimb unloading of neonatal rats inhibited the learning capacity, which was tested by using water maze. Acute tail suspension depressed brain's learning ability and quality, while one-week suspension impaired the spatial memory in mice [50]. Furthermore, it was reported that hindlimb suspension of newly weaned rats caused a decrease of the number of immature doublecortin-positive neurons without any changes in the number of proliferating cell nuclear antigen-positive cells in the dentate gyrus of rats [51].

Further, deafferentaion at L5 segmental region of spinal cord caused significant decrease of protein expressions in thalamus and hypothalamus of rats (unpublished observation). These results suggest that leg exercise or afferent input plays an important role in the regulation of brain properties. The responses of proteins to spaceflight alone were greater than those to housing in MDS on the ground (data not shown), in which the moving area is limited two dimensionally (1.6×9.8 cm) on the floor. However, the moving area for each mouse in MDS is increased three dimensionally to 11.6×9.8×8.4 cm in microgravity environment, although the antigravity muscular activities are inhibited [15], [52], [53]. Furthermore, effects of hindlimb suspension and exposure to 2-G environment, which were performed as the post-flight control experiments on the ground for 3 months using the mice with the same strain, age, and sex were less than the effects seen after spaceflight (unpublished observation). Therefore, these data suggest that greater responses of gene and protein expression to long-term spaceflight may not be related to simply inhibited activities of hindlimb muscles and afferent input. It is speculated that exposure to radiation in space maybe an additional factor for such responses of brain properties. Moreover, compensatory and protective pathways have been reported to be activated in specific muscle in order to counteract microgravity-induced atrophy [54]. Behavioral observations also indicated an increased time spent for “energy saving” behavior in several mice [20]. However, the precise response of brain function to long-term exposure to microgravity environment is not clear yet, since statistically significant up- and down-regulations of the same biological processes (metabolic and oxidation-reduction process) and molecular functions (catalytic activity and oxidoreductase activity), were observed, for example (Table 1 and 2).

The data obtained in the present investigation, even though coming from a small number of animals, suggested that spaceflight caused a modification of numerous genes and proteins in the brain and appears to interfere with expression of neuropeptides involved in psycho-neuro-endocrine adaptations. It was also indicated that the mouse might be a good model for future space biology research aiming to investigate the consequence of the adaptation to different gravity environments.

## Supporting Information

Table S1 **Genes up-regulated by spaceflight (vs. ground control).**
(XLS)

Table S2 **Genes down-regulated by spaceflight (vs. ground control).**
(XLS)
